# Acute Disseminated Encephalomyelitis Following Nivolumab Therapy for Hodgkin Lymphoma: A Case Report and Literature Review

**DOI:** 10.7759/cureus.114593

**Published:** 2026-08-16

**Authors:** Amal Abudouleh, Malak Sondoqah, Areen Said

**Affiliations:** 1 College of Medicine, University of Sharjah, Sharjah, ARE; 2 Department of Neurology, American Hospital Dubai, Dubai, ARE

**Keywords:** acute disseminated encephalomyelitis, autoimmune encephalitis, central nervous system demyelination, hodgkin lymphoma, immune checkpoint inhibitors, neurological immune-related adverse events, nivolumab

## Abstract

The development of immune checkpoint inhibitors (ICIs) such as nivolumab, a programmed death receptor-1 (PD-1) inhibitor, has transformed the management of numerous malignancies. However, they are associated with multiple immune-related adverse events (irAEs), including central nervous system (CNS) demyelination such as acute disseminated encephalomyelitis (ADEM). We report a case of ADEM following nivolumab monotherapy and review the literature on similar presentations.

We present the case of a patient with Hodgkin lymphoma who presented with confusion, disorientation, and seizures following nivolumab therapy. Serial magnetic resonance imaging (MRI) revealed the development of multifocal white matter lesions suggestive of demyelination. The clinical picture, coupled with extensive investigations, led to a diagnosis of ADEM. The patient was managed with intravenous (IV) methylprednisolone, IV immunoglobulin (IVIG), and plasmapheresis and showed clinical improvement. A review of the literature yielded only a few examples of nivolumab monotherapy-induced autoimmune encephalitis and ADEM. The overlap in the clinical presentations of those neurological adverse events could make the diagnosis more challenging.

ICIs are associated with several CNS irAEs, such as ADEM. This case highlights the need for high clinical vigilance for ADEM, an ICI-related CNS complication. Serial imaging and exclusion of alternative etiologies are essential for timely diagnosis and management. Patient outcomes may be improved by early detection and intervention.

## Introduction

The development of immune checkpoint inhibitors (ICIs) has led to significant advances in cancer treatment. Nivolumab, a programmed death receptor-1 (PD-1) inhibitor and a type of ICI, is used in the treatment of multiple malignancies, including Hodgkin lymphoma [1,2]. However, despite their numerous clinical advantages, ICIs can lead to many immune-related adverse events (irAEs) [3]. irAEs commonly affect the gastrointestinal, endocrine, and dermatological systems. However, neurological complications remain relatively rare [4,5].

According to the literature, nivolumab-related neurotoxicity can present as myasthenia gravis, encephalitis [6], or other peripheral or central nervous system (CNS) manifestations. Although rare, acute disseminated encephalomyelitis (ADEM) has been reported as an adverse event associated with ICIs [7]. ADEM refers to an inflammatory demyelinating disorder affecting the CNS and has traditionally been linked to infections and immunizations [8]. Because ADEM and other ICI-related inflammatory CNS disorders, particularly autoimmune encephalitis, may present with overlapping clinical features, distinguishing between these entities can be challenging.

In this article, we discuss the case of a patient who developed ADEM after the initiation of nivolumab therapy. We aim to expand the clinical knowledge about ADEM, a rare but serious neurological toxicity associated with nivolumab, while highlighting the importance of early detection and prompt therapy to improve clinical outcomes and guide future research.

## Case presentation

We present the case of an adult patient with advanced classical Hodgkin lymphoma who was receiving first-line Nivo-AVD therapy, consisting of nivolumab in combination with doxorubicin (Adriamycin), vinblastine, and dacarbazine. Nivolumab was administered intravenously at a dose of 240 mg every two weeks. At the time of neurological presentation, the patient had been receiving first-line Nivo-AVD therapy for approximately four months. The patient had a history of psoriasis but no prior history of neurological disease, seizures, or demyelinating disorders.

Several months after initiating nivolumab therapy, the patient developed acute neurological symptoms, including jaw clenching, teeth tightness, forgetfulness, disorientation, and subsequently a generalized tonic-clonic seizure. Upon arrival to the emergency department, the patient was postictal, confused, afebrile, and without neck stiffness. The initial differential diagnosis included viral encephalitis, autoimmune encephalitis, metabolic encephalopathy, and seizures secondary to structural brain lesions.

During admission, diagnostic investigations were performed and are summarized in Table 1. Brain magnetic resonance imaging (MRI) was unremarkable. Electroencephalography (EEG) demonstrated generalized background slowing. Lumbar puncture (LP) revealed inflammatory cerebrospinal fluid (CSF) findings, including pleocytosis and elevated protein. Blood cultures, CSF cultures, and CSF herpes simplex virus (HSV) polymerase chain reaction (PCR) were negative. Serum and CSF autoimmune and paraneoplastic evaluations were also unrevealing, including negative MOG-IgG testing in both serum and CSF.

**Table 1:** Summary of diagnostic investigations *Obtained during the second admission. **Obtained during the third admission. MRI: magnetic resonance imaging; EEG: electroencephalography; LP: lumbar puncture; WBC: white blood cell; RBC: red blood cell; CSF: cerebrospinal fluid; HSV: herpes simplex virus; PCR: polymerase chain reaction; CNS: central nervous system

Test name	Result	Normal range	Interpretation
MRI of the brain	Unremarkable	No lesions	No acute structural lesion
EEG	Generalized background slowing	Normal EEG background	Diffuse cerebral dysfunction consistent with encephalopathy
LP: WBC	49 cells/mm³ (90% polymorphonuclear cells)	0-5 cells/mm³	Pleocytosis due to inflammation
LP: RBC	7,000 cells/mm³	0 cells/mm³	Traumatic tap
LP: protein	0.92 g/L	0.15-0.45 g/L	Inflammation-induced disruption of the blood-brain barrier
Blood cultures	Negative	Negative	No bloodstream infection identified
CSF cultures	Negative	Negative	No infectious etiology identified
CSF HSV PCR	Negative	Negative	HSV infection excluded
Autoimmune and paraneoplastic panels	Negative	Negative	No specific autoimmune or paraneoplastic antibodies identified
Repeat MRI*	Multifocal white matter lesions	No lesions	Multifocal inflammatory/demyelinating CNS lesions
CSF oligoclonal bands**	Positive	Negative	Indicates intrathecal antibody production, supporting CNS inflammation

Given the CSF pleocytosis and acute neurological presentation, empiric antimicrobial therapy with acyclovir, ampicillin, and vancomycin was initiated while awaiting infectious workup results. Antimicrobials were discontinued after infectious etiologies were excluded. The patient showed clinical improvement and was discharged on levetiracetam 1 g twice daily for seizure prophylaxis.

**Figure 1:**
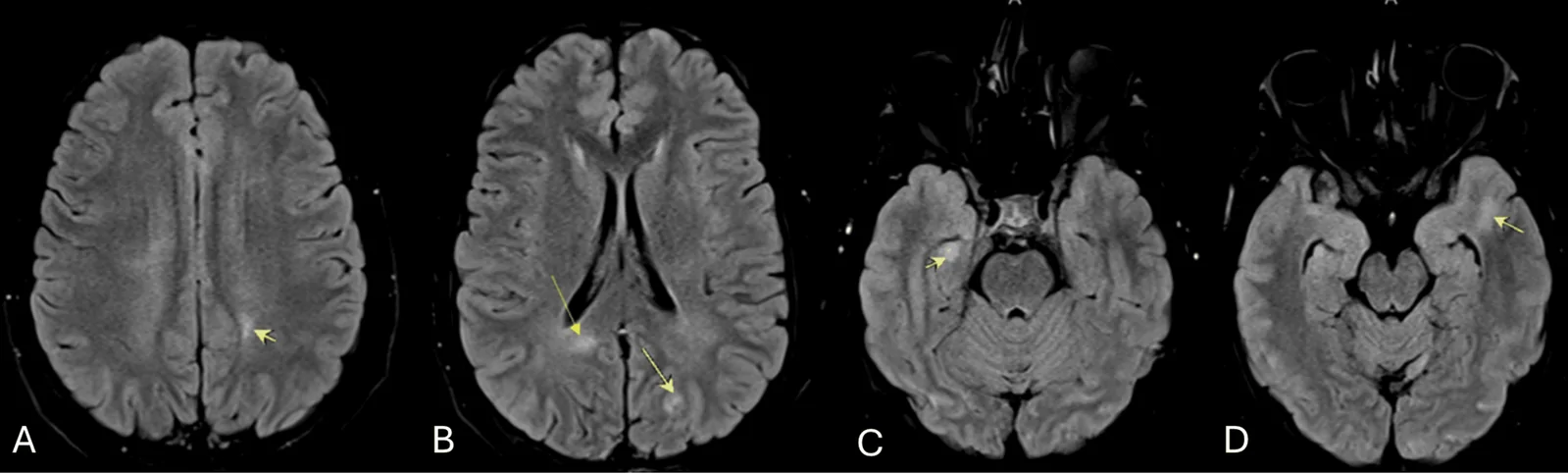
Brain MRI demonstrating multifocal demyelinating lesions FLAIR MR images at multiple brain levels demonstrate multifocal white matter lesions consistent with a central nervous system demyelinating process (A-D). The yellow arrows indicate the multifocal demyelinating lesions. MRI: magnetic resonance imaging; FLAIR: fluid-attenuated inversion recovery

A few days later, the patient returned with fluctuating hearing loss, tinnitus, speech disturbance, perioral paresthesia, confusion, and recurrent focal seizures evolving into bilateral tonic-clonic seizures. As demonstrated in Figure 1, repeat MRI revealed multifocal white matter lesions consistent with a CNS demyelinating process and suggestive of ADEM. Repeat EEG demonstrated epileptiform activity with spike components over the right frontal region. Repeat LP revealed 11 WBCs/mm³ (26% polymorphonuclear cells and 75% lymphocytes) and 7,755 RBCs/mm³. Antiepileptic therapy was adjusted, with levetiracetam increased to 1.5 g twice daily and lacosamide initiated at 100 mg twice daily.

Given the temporal association with ICI therapy, the development of multifocal white matter lesions on serial MRI, and the exclusion of infectious etiologies, an inflammatory/demyelinating CNS process was favored, with ADEM considered the most likely diagnosis.

High-dose intravenous (IV) methylprednisolone (1 g/day) and IV immunoglobulin (IVIG) (0.4 g/kg/day for five days) were initiated. Shortly thereafter, lacosamide was discontinued due to bradycardia, which resolved promptly, and perampanel was added. The patient completed the corticosteroid and IVIG courses, demonstrating significant neurological improvement, and was discharged with a tapering regimen of oral prednisolone.

**Figure 2:**
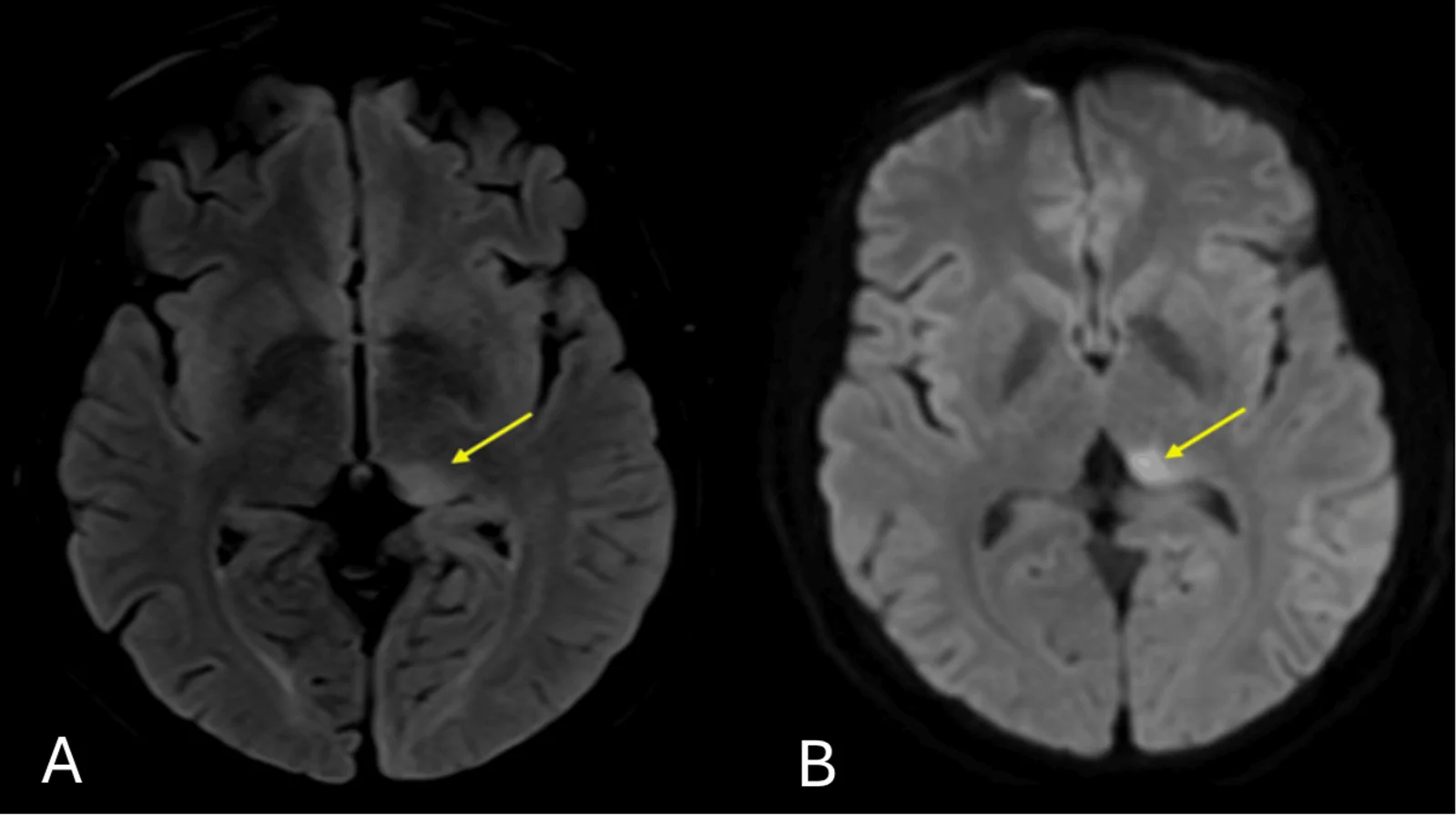
Left thalamic lesion on follow-up MRI FLAIR (A) and DWI (B) MRI sequences demonstrate the development of a new left thalamic lesion. The yellow arrow indicates the left thalamic lesion. MRI: magnetic resonance imaging; FLAIR: fluid-attenuated inversion recovery; DWI: diffusion-weighted imaging

Two weeks after the second discharge, the patient presented with multiple intractable seizures. EEG demonstrated lateralized discharges over the left temporoparietal region. Repeat LP was largely unremarkable, showing 4.9 WBCs/mm³, 17 RBCs/mm³, and protein of 0.41 g/L; however, oligoclonal bands were detected. Additionally, as shown in Figure 2, follow-up MRI demonstrated interval development of a new left thalamic lesion, which was hyperintense on T2-weighted and FLAIR sequences and demonstrated diffusion restriction. No pathological gadolinium enhancement was identified. Additional multifocal bilateral asymmetric white matter lesions were noted, without evidence of leptomeningeal enhancement.

The patient was subsequently restarted on high-dose IV methylprednisolone and underwent five sessions of plasmapheresis, resulting in marked seizure control and neurological improvement. Although initially adherent to treatment, levetiracetam and perampanel were discontinued due to intolerance manifested by behavioral changes, including reported self-harm ideation. Brivaracetam and valproic acid were subsequently initiated at 100 mg twice daily and 500 mg twice daily, respectively.

Follow-up MRI demonstrated regression of the left thalamic lesion and improvement of other previously affected areas. No adverse or unanticipated events were observed during the remainder of treatment. At the last follow-up, the patient remained clinically stable with no further seizure activity.

## Discussion

We present a case of ADEM in an adult treated with nivolumab, highlighting a rare but serious neurological irAE associated with ICI therapy.

ICIs have transformed the management of Hodgkin lymphoma and other malignancies. Nivolumab in combination with AVD (Nivo-AVD) is now an established upfront treatment option for advanced-stage classical Hodgkin lymphoma [9]. However, disruption of immune tolerance can result in a broad spectrum of immune-mediated toxicities, including CNS involvement [4,6,7].

Although ADEM classically occurs following infections or vaccinations, rare cases have also been reported in association with ICIs [7,8]. Our case highlights the importance of recognizing ADEM as a rare but potentially life-threatening neurological complication occurring temporally in association with nivolumab therapy and requiring prompt diagnosis and management.

Nivolumab is a fully human immunoglobulin G4 (IgG4) monoclonal antibody that binds to PD-1 and blocks its interaction with PD-L1 and PD-L2, thereby enhancing antitumor immune activity [10]. The exact mechanism underlying ICI-associated ADEM remains incompletely understood; however, it is thought to involve the disruption of peripheral immune tolerance and dysregulated T-cell activation. PD-1 inhibition may promote autoreactive T-cell proliferation, which in susceptible individuals could target myelin antigens and trigger demyelination [11]. Our patient's history of autoimmune comorbidities may suggest an underlying tendency toward immune dysregulation, although its contribution to the development of CNS autoimmunity remains uncertain.

Although standardized diagnostic criteria for ADEM in adults remain less well established than in children, a diagnosis of ADEM was supported by the acute encephalopathic presentation with multifocal neurological manifestations, inflammatory CSF findings, evolution of multifocal white matter abnormalities on serial MRI, and exclusion of infectious, autoimmune, paraneoplastic, and structural etiologies [12]. Notably, the initial MRI was unremarkable, whereas subsequent imaging demonstrated multifocal white matter lesions followed by the development of a new left thalamic lesion with diffusion restriction. This evolution highlights that early neuroimaging may be nondiagnostic and that repeat MRI can be important when clinical suspicion for an inflammatory demyelinating process remains high. 

Existing research on nivolumab-associated neurological toxicities includes examples of encephalitis, chronic inflammatory demyelinating polyneuropathy, and paraneoplastic syndromes, but ADEM remains extremely rare [13]. Among reported neurological irAEs, encephalitis accounts for 13% of cases, whereas CNS demyelinating diseases account for 2% [14]. Encephalitis and CNS demyelinating diseases such as ADEM may have similar symptoms at presentation, including cognitive impairment, mental status changes, and seizures. Paraclinical investigations, including MRI and autoimmune antibody testing, can aid in differentiating between these entities. Although temporal lobe involvement is common in some forms of autoimmune encephalitis, ADEM is more typically characterized by multifocal demyelinating lesions on MRI [13,14]. The presence of neuronal autoantibodies associated with autoimmune encephalitis may support that diagnosis, whereas AQP4-IgG and MOG-IgG testing can help evaluate alternative inflammatory demyelinating disorders [15]. In our patient, the development of multifocal white matter lesions on serial MRI, together with negative autoimmune and paraneoplastic evaluations, favored a demyelinating process such as ADEM over autoimmune encephalitis.

Given the clinical overlap between ICI-associated autoimmune encephalitis and ADEM, we performed a targeted Google Scholar search using the terms "Nivolumab" AND "Monotherapy" AND "Encephalitis" to identify relevant neurological presentations for comparison. We identified 20 relevant nivolumab-associated neurological cases, which are summarized in Tables 2-3. These cases were included to illustrate the diagnostic challenges in distinguishing autoimmune encephalitis from ADEM.

**Table 2:** Clinical summary of nivolumab-associated encephalitis and ADEM cases All cases listed in the tables are extracted from published case reports of autoimmune encephalitis, except for the case described by Zafar et al. [7] and our own, which are ADEM. ADEM: acute disseminated encephalomyelitis

Study	Patient age	Cancer type	Drug dose/onset of symptoms	Symptoms
Our study	Adult	Hodgkin lymphoma	Nivolumab (240 mg IV)/four months after the first dose	Speech disturbances, jaw clenching, hearing loss, tinnitus, perioral and hand paresthesia, breathing difficulty, and left-sided tonic seizures
Mysler et al., 2023 [16]	67/F	Clear cell renal carcinoma	Nivolumab	Confusion
AlSabah and Altman, 2023 [17]	40/F	Posterior scalp melanoma	Nivolumab, dose not specified/one month after the first cycle	Acute confusion, short-term memory loss, and bilateral tonic-clonic seizures
Costa e Silva et al., 2021 [18]	70/M	Squamous cell carcinoma of the lung	Nivolumab 240 mg every 2 weeks/after 22 doses of nivolumab	Fever, altered consciousness, aphasia, and paratonia
Bross et al., 2020 [19]	70/M	Metastatic clear renal cell carcinoma	Nivolumab dose not specified/onset of symptoms not specified	Toni- clonic seizure and left visual field deficit
Guidi et al., 2020 [20]	60/F	Squamous cell carcinoma of the right tonsil	Nivolumab 240 mg every 2 weeks/after 19 weeks (9 infusions in total)	Progressive altered consciousness, drowsiness, confusion, and coma
Erol-Yildiz et al., 2020 [21]	33/ F	Hodgkin lymphoma	Nivolumab, dose not specified/57th day of treatment (after 4 doses)	Behavioral disturbance, visual hallucinations, agitation, delusion, memory impairment, and seizures
Taillefer et al., 2020 [22]	68/M	Stage IV BRAF-unmutated ocular melanoma with subcutaneous metastasis	IV nivolumab 3 mg/kg every 2 weeks/after the ninth cycle (123 days after the first dose)	Articular and muscular pain, weakness, confusion, and memory impairment
Cabral et al., 2020 [23]	75/F	Non-small cell lung cancer	Nivolumab (480 mg/month)/4 weeks after treatment	Transient episode of aphasia and right-sided sensory disturbance with subacute cognitive decline
Zafar et al., 2019 [7]	59/F	Laryngeal squamous cell carcinoma	Nivolumab, 3 mg/kg every 2 weeks/12 days after the first dose	Gradual weakness, confusion, and progressive dyspnea
Nalbantoğlu et al., 2021 [24]	40/M	Hodgkin lymphoma	Nivolumab dose not specified/one month after the first dose	Hand tremor, confusion, and imbalance
Burke et al., 2018 [25]	64/F	Clear cell ovarian cancer	Nivolumab, dose not specified/after 8 doses	Fever and delirium
Chaucer et al., 2018 [26]	44/M	Renal cell carcinoma	Nivolumab, 240 mg/2 weeks after the first dose	Altered mental status
Shah et al., 2018 [27]	66/F	Lung adenocarcinoma	Nivolumab, dose not specified/after 4 months of treatment	Right hemiballismus and dysarthria
Shah et al., 2018 [27]	44/F	Stage IV lung adenocarcinoma	Nivolumab, 3 mg/kg, every 2 weeks/after 5 cycles	Progressive altered mental status, nausea, vomiting, and seizures
De la Hoz et al., 2019 [28]	28/M	Hodgkin lymphoma	Nivolumab 80 mg on day 1; increased to 120 mg (2 mg/kg) on day 14/after the second dose	Worsening frontal headache with nausea and dizziness, apathy, and mild impaired consciousness
Matsuoka et al., 2018 [29]	60/M	Stage IV pleomorphic carcinoma of the lung	Nivolumab 3 mg/kg, every 2 weeks/5 days after the third dose of nivolumab	Drowsiness and memory disturbance progressing to muscular weakness on day 16
Kopecký et al., 2018 [30]	63/M	Metastatic renal cell cancer	Nivolumab (300 mg every 14 days)/14 days after the final dose	Altered behavior, abnormal movements, and restlessness
Richard et al., 2017 [31]	74/M	Stage IV squamous non-small cell lung cancer	Nivolumab dose not specified/within one week	Progressive altered mental status
Schneider et al., 2017 [32]	78/M	Squamous cell carcinoma of the lung	Nivolumab, 3 mg/kg every 2 weeks/12 days after the 14th cycle of nivolumab (28 weeks after the first dose)	Apathy and aphasia
Larkin et al., 2017 [33]	83/F	Advanced melanoma	Nivolumab, 3 mg/kg every 2 weeks/after four doses	Altered mental status

**Table 3:** Management summary of nivolumab-associated encephalitis and ADEM cases All cases listed in the tables are extracted from published case reports of autoimmune encephalitis, except for the case described by Zafar et al. [7] and our own, which are ADEM. ADEM: acute disseminated encephalomyelitis; MRI: magnetic resonance imaging; RBCs: red blood cells; WBCs: white blood cells; CSF: cerebrospinal fluid; IVIG: intravenous immunoglobulin; PCR: polymerase chain reaction; +ve: positive; -ve: negative

Study	MRI findings	CSF findings	Treatment	Outcome
Our study	Hyperintense lesions in the left thalamus with diffusion restriction	Pleocytosis, high RBCs, and normal glucose levels. High protein. +ve oligoclonal bands. -ve cytology, culture, and PCR. -ve autoantibodies	IV methylprednisolone (1 g/day) for 5 days, followed by IVIG therapy (2 g/kg) over 5 days	Clinical improvement
Mysler et al., 2023 [16]	Hyperintensity in posterior periventricular white matter bilaterally	Pleocytosis and normal RBCs and glucose levels. High protein. -ve oligoclonal bands. Cytology not reported. -ve culture and PCR. -ve antibodies	IV methylprednisolone (1 g/day) for 5 days	Clinical improvement
AlSabah and Altman, 2023 [17]	Hyperintense lesions in the temporal lobe involving the hippocampus with diffusion restriction	Pleocytosis and normal RBCs and glucose levels. Normal protein. Oligoclonal bands not reported. Cytology not reported. Negative cultures and PCR. +ve antineuronal antibody	IV methylprednisolone (1 g) for 5 days, followed by IVIG for 5 days and 5 plasma exchanges	Lack of clinical improvement noted leading to the initiation of natalizumab. Thereafter, due to high JC antibody index, natalizumab was discontinued, and cyclophosphamide was started
Costa e Silva et al., 2021 [18]	Mild enhancement in meninges	Pleocytosis and normal RBCs and glucose. High protein. Oligoclonal bands not reported. Cytology, culture, and PCR not reported. Autoantibodies not reported	IV methylprednisolone (1 g/day) for 5 days, followed by IVIG therapy (0.4 g/kg/day) for 5 days	Clinical improvement (after 1 day of treatment)
Bross et al., 2020 [19]	Solid-enhancing right occipital mass (inflammatory tumor-like lesion)	Pleocytosis and normal RBCs and glucose. Normal protein. Oligoclonal bands not reported. -ve cytology, cultures, and PCR. Autoantibodies not reported	Steroids (dose not specified)	Clinical improvement
Guidi et al., 2020 [20]	Hyperintense lesions in the posterior, frontal-medial, parietal, and occipital regions	Normal	IV methylprednisolone (120 mg (3 mg/kg/d) bid)	Clinical improvement (after 3 days of treatment)
Erol-Yildiz et al., 2020 [21]	Unremarkable	Normal WBCs, RBCs, and glucose. Normal protein. Oligoclonal bands not conducted. -ve cytology and PCR. Culture not reported. -ve autoantibodies	IV methylprednisolone (1 g/day) for 5 days, followed by IVIG (0.4 g/kg/day) for 5 days	Clinical improvement (after 21 days of treatment)
Taillefer et al., 2020 [22]	Hyperintense lesion in the left temporal lobe	Normal WBCs, RBCs, and glucose. High protein. -ve oligoclonal bands. -ve cytology, culture, and PCR. -ve autoantibody	IV methylprednisolone 1 g for 5 days	Clinical improvement
Cabral et al., 2020 [23]	Unremarkable	Pleocytosis and normal RBCs and glucose. High protein. -ve oligoclonal bands. -ve cytology, culture, and PCR. -ve autoantibodies	Prednisolone 1 mg/kg/day	Clinical improvement (over 2 months of treatment)
Zafar et al., 2019 [7]	Hyperintense white matter lesions in the posterior frontal lobes, corpus callosum, and right brachial pontis	Pleocytosis. Normal RBCs and glucose. High protein. +ve oligoclonal bands. -ve cytology, culture, and PCR. Autoantibodies not reported	IV methylprednisolone (1 g/day) for 5 days, followed by IVIG(20 g/day) for 4 days	Clinical improvement (after 12 days of treatment)
Nalbantoğlu et al., 2021 [24]	Enhancing millimetric lesions in the left frontal and right occipital lobes	Pleocytosis and normal RBCs and glucose. High protein. +ve oligoclonal bands. -ve cytology, culture, and PCR. -ve autoantibodies	IV methylprednisolone 1g daily for 10 days followed by oral methylprednisolone 32 mg daily for one month	Clinical improvement (after 1 month of treatment)
Burke et al., 2018 [25]	Unremarkable	Unremarkable	IV methylprednisolone (3g, 4×/day), plasmapheresis (10 times), and antibiotics	Clinical improvement
Chaucer et al., 2018 [26]	No metastatic lesions or lymphoreticular disorder findings	Not reported	Haldol for agitation, ketamine for sedation, Precedex for hypertension	Clinical improvement
Shah et al., 2018 [27]	Symmetric T2 hyperintense and T1 hypointense basal ganglia abnormalities	Normal WBCs, RBCs, and glucose. High protein. +ve oligoclonal bands. -ve cytology. Culture and PCR not reported. +ve autoantibodies (not specified)	IV methylprednisolone (1 g/day) for 5 days, 5 plasma exchanges, IVIG (total of 2.5 g/kg), prednisone, rituximab (1 g once), and tetrabenazine (20 mg, 3×/day)	Lack of clinical improvement leading to transition to inpatient hospice and comfort-only care
Shah et al., 2018 [27]	Hyperintense lesions of the bilateral mesial temporal lobes	Pleocytosis and normal RBCs and glucose. Normal protein. +ve oligoclonal bands. -ve cytology. Culture and PCR not reported. +ve autoantibodies (GAD65)	IV methylprednisolone (1 g/day) for 5 days, 5 plasma exchanges, and rituximab (1 g) (for refractory seizures)	Clinical improvement
De la Hoz et al., 2019 [28]	Unremarkable	Pleocytosis and normal RBCs and glucose. High protein. Oligoclonal bands not reported. Cytology not reported. Normal culture and PCR. Autoantibodies not reported	IV methylprednisolone 1 mg/kg daily	Clinical improvement (after 1 day of treatment)
Matsuoka et al., 2018 [29]	High-intensity areas in the temporal lobe, thalamus, cerebral aqueduct, and spinal cord	Pleocytosis and normal RBCs and glucose. High protein. +ve oligoclonal bands. -ve cytology, culture, and PCR. CSF autoantibodies not reported. +ve serum anti-Hu antibody	High-dose methylprednisolone (×2, dose not specified) and plasmapheresis (×2)	The patient died from lung cancer, and autopsy revealed positive anti-Hu antibodies
Kopecký et al., 2018 [30]	Symmetric hyperintensities in the basal ganglia	Pleocytosis. RBCs, glucose, and protein not reported. -ve cytology, culture, and PCR. +ve autoantibodies (anti-MA2 IgG)	IV methylprednisolone 2 mg/kg per day. Infliximab 5 mg/kg was started due to deterioration despite therapy	The patient left the hospital after signing an against medical advice discharge form. Three weeks later, the patient was readmitted and died 4 days after admission
Richard et al., 2017 [31]	Not conducted	Normal glucose. WBCs and RBCs not reported. Normal protein. Oligoclonal bands not reported. -ve cytology, culture, and PCR. Autoantibodies not reported	IV methylprednisolone on day 5	Clinical improvement (after 3 days of treatment)
Schneider et al., 2017 [32]	No metastasis, meningitis, or stroke	Pleocytosis, normal RBCs, and low glucose. High protein. Oligoclonal bands not reported. -ve cytology, culture, and PCR. Autoantibodies not reported	Methylprednisolone (1.33 mg/kg body weight)	Clinical improvement
Larkin et al., 2017 [33]	Hyperintense lesions in the supratentorial white matter	Normal WBCs and glucose. High protein. Oligoclonal bands not reported. -ve culture. Cytology and PCR not reported. Autoantibodies not reported	Maintenance prednisone 30 mg/day	Lack of clinical improvement followed by death

To our knowledge, only one previous case of ADEM associated with nivolumab monotherapy has been reported. This case, described by Zafar et al. [7], involved a patient with progressive laryngeal squamous cell carcinoma treated with nivolumab (3 mg/kg) who developed weakness, confusion, and dyspnea 12 days after the initial dose. MRI revealed multifocal cerebral demyelination consistent with ADEM. Discontinuation of nivolumab, along with IV methylprednisolone (1 g/day for five days) followed by IVIG (20 g/day for four days), resulted in clinical improvement.

The documented cases range in age from 28 to 83 years. Our case, involving a patient with Hodgkin lymphoma, is consistent with previously reported cases in relatively young patients who developed encephalitis during nivolumab therapy [21,24,28]. However, the limited number of cases precludes conclusions regarding age- or cancer-specific susceptibility.

Most reported patients received standard nivolumab regimens (240 mg flat dose every two weeks or 3 mg/kg every two weeks), consistent with current guidelines. One study used dose escalation [28], but no clear dose-response pattern emerged. Our patient received the standard regimen, highlighting that irAEs can occur even with standard dosing methods.

The timing of neurological toxicity following nivolumab exposure is variable. In a pooled analysis of patients with advanced melanoma receiving nivolumab monotherapy or nivolumab with ipilimumab, the median time to onset of serious neurological irAEs was 45 days, with a reported range of 1-170 days [33]. Similarly, the nivolumab-associated cases included in our review demonstrated considerable variability in symptom onset, ranging from the first few weeks to several months after treatment initiation. In our case, symptoms developed after several months of nivolumab therapy, further highlighting the variable timing of these neurological adverse events.

Most cases demonstrated MRI abnormalities, commonly involving hyperintense lesions in the white matter or deep gray matter. However, some individuals had unremarkable imaging, suggesting that radiological findings can be variable or even absent. CSF findings were more consistent, with pleocytosis and elevated protein commonly reported, while glucose was often normal. Oligoclonal bands and autoantibodies were variably detected, underscoring the diagnostic challenges associated with nivolumab-related neurological toxicity.

Most patients improved following treatment with high-dose IV methylprednisolone, although some required additional therapies, including IVIG, plasmapheresis, or immunosuppressive agents. Our patient initially demonstrated clinical improvement following corticosteroids and IVIG; however, recurrent seizures and a new left thalamic lesion subsequently developed, prompting plasmapheresis. The absence of detectable autoimmune antibodies may have been associated with the favorable clinical response; however, further studies are needed to determine the predictors of treatment response.

Strengths and limitations

Our case report provides a detailed clinical timeline and comprehensive diagnostic evaluation, adding to the limited literature on nivolumab-associated ADEM. By documenting both diagnostic challenges and therapeutic decision-making, this report may offer valuable guidance for clinicians managing patients with similar presentations.

Despite these strengths, this case report has several limitations, including its single-patient design, which limits generalizability and precludes the definitive establishment of a causal relationship between nivolumab and ADEM. The lack of extended follow-up also limits conclusions regarding long-term outcomes.

Clinical implications and future directions

This case underscores the importance of maintaining clinical vigilance in patients receiving ICIs who present with neurological symptoms. Early MRI and CSF analysis are crucial in distinguishing ICI-associated neurotoxicity from infectious or paraneoplastic etiologies [11,14]. A history of pre-existing autoimmune disease may also warrant consideration during clinical assessment, although its relationship with the risk of neurological irAEs remains uncertain. Larger case series are needed to better understand the pathophysiology of ICI-associated ADEM and to guide standardized management strategies.

## Conclusions

This report describes a case of ADEM temporally associated with nivolumab therapy for Hodgkin lymphoma. Although rare, CNS demyelinating disorders should be considered in patients receiving ICIs who develop new neurological symptoms. In this case, treatment with high-dose corticosteroids, IVIG, and plasmapheresis was followed by clinical and radiological improvement. Early recognition and prompt management may be important in improving outcomes in ICI-associated neurological toxicity.
